# Nontuberculous Mycobacterial Infection Is Associated with Increased Respiratory Failure: A Nationwide Cohort Study

**DOI:** 10.1371/journal.pone.0099260

**Published:** 2014-06-11

**Authors:** Jun-Jun Yeh, Yu-Chiao Wang, Cheng-Li Lin, Christine Yi-Ting Chou, Ting-Chun Yeh, Bing-Tsang Wu, Fung-Chang Sung, Chia-Hung Kao

**Affiliations:** 1 Ditmanson Medical Foundation Chia-Yi Christian Hospital, Chiayi, Taiwan; 2 Chia Nan University of Pharmacy and Science, Tainan, Taiwan; 3 Meiho University, Pingtung, Taiwan; 4 Management Office for Health Data, China Medical University Hospital, Taichung, Taiwan; 5 Graduate Institute of Clinical Medical Science and School of Medicine, College of Medicine, China Medical University, Taichung, Taiwan; 6 UCSD Medical Center-Hillcres, San Diego, California, United States of America; 7 Department of Nuclear Medicine and PET Center, China Medical University Hospital, Taichung, Taiwan; National Institute of Infectious Diseases, Japan

## Abstract

**Background and Purpose:**

Population study on relationship between nontuberculous mycobacterial (NTM) infection and respiratory failure (RF) is limited. This study evaluated the RF risk, including acute respiratory failure (ARF), chronic respiratory failure (CRF) and ARF on CRF, in patients with NTM infection in Taiwan.

**Methods:**

We used the National Health Insurance Research Database of Taiwan to identify 3864 newly diagnosed NTM patients (NTM cohort) from 1999 to 2009, and 15456 non-NTM patients (non-NTM cohort), frequency matched by demographic status for comparison. Incidence and hazard of developing RF were measured by the end of 2010.

**Results:**

The incidence rate of RF was 4.31-fold higher in the NTM cohort than in the non-NTM cohort (44.0 vs.10.2 per 1000 person-years), with an adjusted hazard ratio (HR) of 3.11 (95% CI: 2.73–3.54). The cumulative proportional incidence of RF was 10% higher in the NTM cohort than in the non-NTM cohort (*P*<0.0001). The RF risk was much greater within 6 months after the diagnosis of NTM infection with a HR of 7.45 (95% CI = 5.50–10.09). Age-specific comparison showed that the younger NTM patients had a higher HR of RF than the elderly NTM patients (HR: 4.42, 95% CI: 3.28–5.96 vs. HR: 2.52, 95% CI: 2.17–2.92). Comorbidity increased the risk of RF in both cohorts, particularly in those with chronic obstructive pulmonary disease.

**Conclusion:**

Our study suggests patients with NTM infection are at a high risk of RF. The risk appears much greater soon after patients diagnosed with NTM infection.

## Introduction

Recent studies have shown that patients with nontuberculous mycobacterial (NTM) infection associated with lung disease are at an increased hospitalization risk [1]–[5]. NTM disease such as mycobacterium avium complex (MAC) related lung disease presents not only in immunocompromised patients with acquired immunodeficiency syndrome (AIDS) [6] but also in human immunodeficiency virus (HIV) negative subjects with no underlying disease [7]. Studies have also demonstrated that the specific radiographic feature may progress over time, which can be confirmed in histopathologic findings of NTM infection [8]. These findings suggest the presence of true infection involving lung tissue invasion in patients with pulmonary NTM disease, rather than colonization even in immunocompetent patients [9]. Meanwhile, Moore et al. suggested that the bronchiectasis in NTM patients with lung lesions is not a preexisting condition but a result from NTM infection [10].

Respiratory failure (RF) occurs as the exchange between O_2_ and CO_2_ fails to meet the need of metabolism, leading to hypoxaemia, with or without hypercarbia. The diagnosis of RF requires to measure arterial blood gases, including the partial pressure of O_2_ (P_a_O_2_) and the partial pressure of CO_2_ (P_a_CO_2_) in the arterial blood. RF can be defined as P_a_O_2_<8 kPa (60 mmHg), or P_a_CO_2_>6.7 kPa (50 mHg) for a patient at rest, breathing air at sea level [11]–[13]. Acute respiratory failure (ARF) develops over several minutes to hours, because of absence of oxygen delivery to the blood or because of an acute failure to remove carbon dioxide (CO_2_) from the blood by the respiratory system. Chronic respiratory failure (CRF) develops over several days or longer [11], [14]. ARF on CRF is owing to acute exacerbation of CRF [11], [14].

RF is a major cause leading to intensive-care for patients requiring hospitalization and mechanical ventilation [4]. The etiology of RF has been associated with tuberculosis, pneumonia, bronchopneumonia, diabetes, chronic obstructive pulmonary disease (COPD), pneumococcosis, end-stage renal disease(ESRD), malnutrition and AIDS [13], [15]–[18]. Several studies also reported that these factors are predisposing factors of NTM infection [1]–[4], [19]–[22]. The NTM infection has been associated with the risk of not only ARF [1], [2] but also CRF [22] in hospitalized patients. No detailed study has addressed the RF risk for inpatients with NTM infection. This retrospective cohort study was designed to use a large population data to evaluate the risk of RF for patients with NTM infection, comparing with the general population [23], [24]. Therefore, we measured the incidence of RF between subjects with and without NTM infection by demographic status and comorbidity using the health insurance claims data of Taiwan.

## Materials and Methods

### Data Source

The National Health Insurance (NHI) program of Taiwan is a universal insurance program established in 1995, reforming from 13 insurance-related systems, and providing health care to 99% of approximately 23 million people in Taiwan. We used the National Health Insurance Research Database (NHIRD) obtained from the insurance system to identify patients and health care providers. The data sets consisted of claim data for outpatients, inpatients, catastrophic illnesses registry and registry of beneficiaries for the period from 1996 to 2010. Patients demographic status including sex, birth date, medical services and medications, and costs were available in these data files. All personal identification numbers had been encrypted before releasing to the public to protect patient privacy. We identified diseases using codes of the International Classification of Diseases, 9^th^ Revision, Clinical Modification (ICD-9-CM) [25]. An ad hoc committee was available in the Bureau of Insurance to monitor the claims. Institutions with violation receive penalty.

### Ethics Statement

Because identification numbers of patients had been encrypted, patient consent was not required for this study. This study was approved by the Research Ethic Committee at China Medical University (CMU-REC-101-012). The committee waived the requirement for consent.

### Study Population

This population-based retrospective cohort study identified a cohort of NTM patients (ICD-9-CM 031.0, 031.2, 031.8, 0.31.9) newly diagnosed from 1999 to 2009 from inpatient claims data. We further randomly selected 15456 individuals free of NTM infection in the reimbursement claim data files as the comparison cohort or non-NTM cohort, frequency-matched by sex, age and index year. The principal outcome was RF (ICD-9-CM 518.81, 518.83, 518.84). Subjects who had experienced RF prior to the baseline year were excluded in both cohorts. Follow-up was terminated by the identification of a RF event or at the end of 2010 or censored for death and loss to follow-up.

Comorbidities with potential association with RF were identified at the baseline [1]–[8], [13]–[22], including tuberculoses (ICD-9-CM 010–018), bronchopneumonia (ICD-9-CM 485), pneumonia (ICD-9-CM 486), diabetes (ICD-9-CM 250), chronic obstructive pulmonary disease (COPD; ICD-9-CM 490–492), liver cirrhosis, (ICD-9-CM 571), end stage renal disease (ESRD; ICD-9-CM 585), human immunodeficiency virus (HIV; ICD-9-CM 042–044, 795.8, V08), pneumoconiosis and other lung diseases due to external agents (ICD-9-CM 500–508) and malnutrition (ICD-9-CM 260–269), identified from inpatient claim data, and cancer (ICD-9-CM 140–208) identified from the catastrophic illness registry. Patients with NTM were further divided into the MAC (ICD-9-CM 031.0, 031.2) and non-MAC (ICD-9-CM 031.8, 0.31.9) subgroups.

### Statistical Analysis

Data analyses used the Chi-square test to examine categorical variables and Student’s *t*-test to assess continuous variables between NTM and non-NTM cohorts for the baseline data. The RF incidence rate was calculated for each cohort. The cumulative proportional incidences of RF were measured using the Kaplan-Meier method for both cohorts during the follow-up period, and examined with the log-rank test. Cox proportional hazards regression was applied to estimate the hazard ratio (HR) and 95% confidence interval (CI) of RF for the NTM cohort, compared with the non-NTM cohort. Data analysis further differentiate the risk between NTM patients with MAC and without (non-MAC).

All statistical analyses were performed using the SAS 9.3 statistical package (SAS Institute Inc., NC, USA). R software (R Foundation for Statistical Computing, Vienna, Austria) was used to construct the Kaplan-Meier curves. A *p-value<*0.05 in 2-tailed tests was considered significant.

## Results

**Figure 1:**
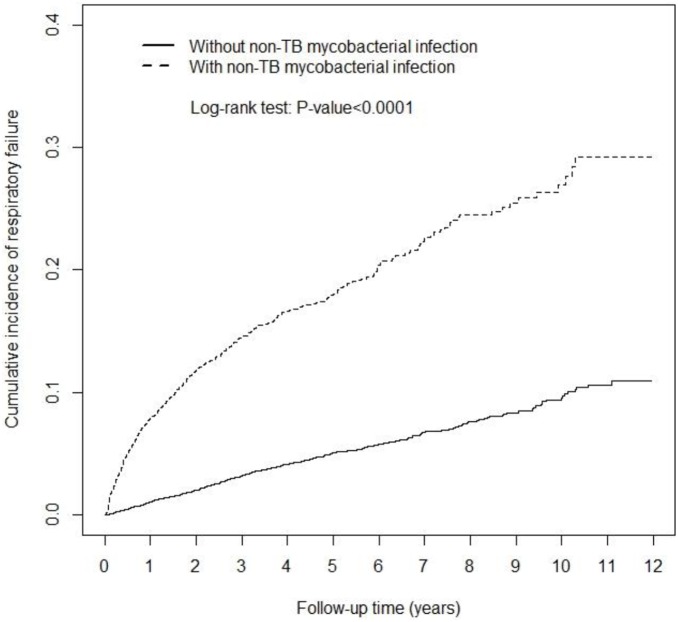
Cumulative incidence of respiratory failure among patients with non-TB mycobacterial infection (dashed line) and among compared subjects.

The study population consisted of 3,864 NTM patients and 15,456 non-NTM comparisons, with similar distributions in sex, age (mean 55.7 year) and occupation (Table 1). The NTM cohort had lower income and were more likely have pre-existing comorbidities than the comparison cohort (*P*<0.001). Kaplan-Meier model estimated cumulative incidence of RF was 10% higher in the NTM cohort than the non-NTM cohort (Figure 1; *P*<0.0001 in the log rank test).

**Table 1:** Comparison of demographics and comorbidities between non-TB mycobacterial infection cohort and comparison cohort.

	Non-TB mycobacterial infection				
	No (N = 15456)		Yes (N = 3864)		
Variables	n	%	N	%	*p*-value
Sex†					0.98
Women	5816	37.63	1454	37.63	
Men	9640	62.37	2410	62.37	
Age, year†					0.98
<65	9388	60.74	2347	60.74	
≥65	6068	39.26	1517	39.26	
Mean (SD)#	55.70 (21.10)		55.73 (21.10)		0.93
Income, NTD†					<.0001
<15000	7456	48.24	2017	52.20	
15000–25000	5057	32.72	1264	32.71	
≥25000	2943	19.04	583	15.09	
Occupation†					0.31
White collar	7467	48.31	1801	46.61	
Blue collar	5890	38.11	1523	39.42	
Others	2047	13.24	527	13.64	
Comorbidity†					
Tuberculosis	169	1.09	900	23.29	<.0001
Bronchopneumonia	128	0.83	154	3.99	<.0001
Pneumonia	511	3.31	795	20.57	<.0001
COPD	583	3.77	766	19.82	<.0001
Pneumoconiosis	70	0.45	101	2.61	<.0001
Diabetes	2505	16.21	748	19.36	<.0001
Liver cirrhosis	505	3.27	316	8.18	<.0001
Cancer	495	3.20	363	9.39	<.0001
ESRD	67	0.43	76	1.97	<.0001
Malnutrition	37	0.24	59	1.53	<.0001
HIV	3	0.02	186	4.81	<.0001

† Chi-square test;

# Student’s t-test.

Table 2 shows that the incidence of RF in the NTM cohort was 4.31-fold higher than that in the comparison cohort (44.0 vs. 10.2 per 1000 person-years), with an adjusted HR of 3.11 (95% CI: 2.73–3.54) (*p*<0.001). The RF incidence was higher in men, the elderly, low income people and subjects with blue collar jobs. Comorbidity increased the risk of RF in both cohorts, particularly in NTM patients with COPD with an incidence of 144.5 per 1000 person-years, which was 5.1-fold greater than the incidence in NTM patients without COPD.

**Table 2:** Incidence of respiratory failure by sex, age, income, occupation and comorbidity and adjusted hazard ratio for patients with non-TB mycobacterial infection compared with comparison subjects without non-TB mycobacterial infection.

	Non-TB mycobacterial infection						Compared to comparisons	
		No			Yes			
Variables	Case	PY	Rate^#^	Case	PY	Rate^#^	IRR^*^(95% CI)	Adjusted HR^†^(95% CI)
All	675	66162	10.20	587	13337	44.01	4.31(4.00–4.65)	3.11(2.73–3.54)
Sex								
Women	206	24811	8.30	164	5298	30.95	3.73(3.28–4.24)	2.67(2.11–3.39)
Men	469	41351	11.34	423	8038	52.62	4.64(4.22–5.10)	3.32(2.84–3.87)
Age, year								
<65	87	43720	1.99	174	9569	18.18	9.14(8.15–10.2)	4.42(3.28–5.96)
≥65	588	22442	26.20	413	3767	109.63	4.18(3.76–4.66)	2.52(2.17–2.92)
Income, NTD^†^								
<15000	459	31229	14.7	375	6477	57.89	3.94(3.55–4.37)	2.88(2.45–3.39)
15000–25000	198	21332	9.28	181	4468	40.51	4.36(3.81–4.99)	3.38(2.68–4.27)
≥25000	18	13601	1.32	31	2391	12.97	9.80(7.86–12.2)	6.36(3.33–12.1)
Occupation								
White collar	219	32666	6.70	225	6478	34.73	5.18(4.62–5.81)	3.47(2.79–4.33)
Blue collar	298	25077	11.88	247	5243	47.11	3.96(3.51–4.47)	2.99(2.45–3.64)
Others	158	8287	19.07	115	1591	72.30	3.79(3.12–4.61)	2.93(2.19–3.91)
Comorbidity								
No	274	52656	5.20	93	7007	13.27	2.55(2.27–2.86)	3.50(2.76–4.43)
Yes	401	13505	29.69	494	6330	78.04	2.63(2.33–2.97)	3.67(3.21–4.20)
COPD								
No	549	64434	8.52	326	11530	28.27	3.32(3.05–3.61)	3.27(2.81–3.81)
Yes	126	1728	72.93	261	1806	144.51	1.98(1.56–2.51)	2.31(1.81–2.94)
Diabetes								
No	440	57077	7.71	416	11181	37.20	4.83(4.43–5.26)	3.50(2.99–4.09)
Yes	235	9085	25.87	171	2155	79.35	3.07(2.59–3.63)	2.33(1.85–2.93)

PY, person-years; Rate^#^, incidence rate, per 1,000 person-years; IRR^*^, incidence rate ratio; CI, confidence interval; Adjusted HR^†^: multivariable analysis included age, sex, income, occupation, and comorbidities.; all p<0.001.

Table 3 shows the trends of RF events by follow-up in both cohorts. The incidence of RF decreased with follow-up time in both cohorts, higher in the NTM cohort than in the non-NTM cohort. The NTM cohort had an adjusted HR of 7.45 (95% CI: 5.50–10.1) (*p*<0.001) in the first 6 months of follow-up.

**Table 3:** Incidence of respiratory failure events in study cohorts by follow-up time and hazard ratio for patients with non-TB mycobacterial infection.

	Without non-TB mycobacterial infection			With non-TB mycobacterial infection				
Follow time	Event	PY	Rate^#^	Event	PY	Rate^#^	IRR^*^ (95% CI	Adjusted HR^†^(95% CI)
<6 months	73	7667	9.52	186	1794	103.69	10.9(9.90–12.0)	7.45(5.50–10.1)
6–12 months	89	7538	11.81	98	1626	60.25	5.10(4.65–5.61)	3.47(2.47–4.86)
1–3 years	263	24208	10.86	186	4873	38.17	3.51(3.22–3.84)	2.49(1.99–3.11)
3–5 years	141	14461	9.75	60	2774	21.63	2.22(1.95–2.52)	1.50(1.04–2.16)
≥5 years	109	12288	8.87	57	2269	25.12	2.83(2.44–3.29)	2.85(2.01–4.04)

PY, person-years; Rate^#^, incidence rate, per 1,000 person-years; IRR^*^, incidence rate ratio; CI, confidence interval; Adjusted HR^†^: multivariable analysis included age, sex, income, occupation, and comorbidities.; p<0.001.

Data analysis further stratified the NTM cohort into MAC and non-MAC subgroups. The incidence of RF was 2.6-fold greater for those with MAC than those without MAC in the NTM cohort (Table 4).

**Table 4:** Incidence and adjusted hazard ratio of respiratory failure associated with mycobacterium avium complex in patients with non-TB mycobacterial infection.

Variables		Event	PY	Rate^#^	IRR^*^(95% CI)	Adjusted HR^†^(95% CI)
Non-TBmycobacterial infection	Comparison cohort	675	66162	10.2	1.00	1.00
	MAC	307	3947	77.8	7.62(6.95–8.36)	3.51(2.98–4.12)
	Non-MAC	280	9390	29.8	2.92(2.66–3.21)	2.85(2.46–3.31)

PY, person-years; Rate^#^, incidence rate, per 1,000 person-years; IRR^*^, incidence rate ratio; CI, confidence interval; Adjusted HR^†^: multiple analysis including age, sex, income and comorbidities; NTM, non-TB mycobacterial infection; MAC, mycobacterium avium complex; Non-MAC, none mycobacterium avium complex; p<0.001.

## Discussion

In the present study, NTM patients exhibited a 4.3-fold higher incidence of RF than the general population, with a HR of 3.11 after adjusting for age, sex, income, occupation, and comorbidities (Table 2). The RF risk increased with age and higher in men than in women. However, the relative HR was higher in younger NTM patients than in the elderly patients (HR: 4.42 vs. 2.52) and higher in men than in women (HR 3.32 vs. 2.67). Lee et al. found a substantial decline in lung function in young NTM patients. [26]. Their findings are consistent with our findings. Therefore, attention should be given to patients with these risk factors.

Comorbidities were more prevalent in the NTM cohort than in the non-NTM cohort in this study. These comorbidities are also risk factors of NTM disease and RF. Among comorbidities, only COPD and diabetes have significant joint effect with NTM for developing RF (see Table S1 in File S1). Huang et al. have found that patients with *M. abscessus* infection without MAC in subtropical chronic ventilatory setting are at a high risk of RF and 39.3% (n = 11) patients with diabetes [3]. On the other hand, Shu et al. have reported that NTM patients are admitted to an intensive care unit mainly with RF (81%), less often with COPD exacerbation (1%), and diabetes was not associated with RF [4]. NTM pulmonary infection may cause an underlying comorbid conditions leading to RF [4], [8]–[10], which in turn worsens the pulmonary function [4], [27]. Meanwhile, the MAC infection can lead to air trapping distal to the small airways for NTM patients [28]. Several studies have found that NTM patients with worse pulmonary test [26], [29] has a higher risk of RF because of comorbidities [1], [2], [5], [30]–[32]. In our study, the multivariate analysis showed that NTM patients with MAC have a much higher incidence of RF than those without MAC. COPD and diabetes further increase the risk of RF.

This study showed that patients diagnosed with NTM within 6 months had the highest risk of RF with an adjusted HR of 7.45 and the HR decreased with follow-up. Shu et al. have reported that the 6-month survival in NTM patients with lung lesion is poor based on a single hospital study. The survival is particularly poor for those with worsening consolidation and cavitation of lung [33]. An earlier study found that 72% of patients with lung consolidation suffered from ARF [4]. Lung destruction appears soon after NTM is diagnosed and the lung function impairment follows [4], [33].

Studies also found that bronchiectasis carries a worse prognosis [33] and develops RF with a relatively slow and indolent course among NTM patients [5], [22], [30]. COPD has a strong association with bronchiectasis in previous study [5]. Meanwhile, NTM is a potential risk factor for COPD [34]. Furthermore, COPD is an important cause of RF [11], [14]. These findings highlight the importance of identifying NTM patients who are likely to have RF.

Studies have found patients with NTM are at a higher risk of fast decline of pulmonary function [33], and the decline of lung function is even greater than patients with COPD [26]. In addition, Ringshausen et al. found 20.4% of patients with the NTM lung disease develop RF [2]. These studies confirm our findings. The cumulative proportional incidence of RF is higher among patients with NTM infection than those without the infection. We have performed additional data analysis to estimated the RF incidence for the 2 study cohorts by the follow-up time of <1 year and ≥1 year with the adjusted HRs of 5.31 (95% CI: 4.26–6.63) and 2.33 (95% CI: 1.97–2.75), respectively, (p<0.0001, Table S2 in File S1). Patients with delayed diagnosis of NTM could develop RF episode and require ICU intervention sooner.

Recent studies have reported the delayed diagnosis of Mycobacterium kansasii septicemia [35], drug resistance [36], and outbreak of nosocomial NTM infection [37] may also contribute to the development of RF in patients with NTM infections. Most NTM patients develop granulomatous inflammation and a large portion have a cavity difficult to distinguish from tuberculosis in the clinical and radiological findings [21]. In countries with a high prevalence of tuberculosis, NTM is likely misdiagnosed as tuberculosis. Thus there is a critical need to improve the diagnostic capacity of mycobacterial disease to highlight the awareness of NTM disease prevalence [35].

A Japanese study found subjects with NTM pulmonary infection but normal underlying lung anatomy developing bronchiectasis because of bronchial wall infection and necrosis similar to tuberculosis [38]. Su et al. have reported that 24 out of 43 patients (56%) with NTM disease without comorbidities were admitted to ICU because of RF [4]. The risk of the RF in the NTM cohort without comorbidities were higher than in the cohort without NTM (HR 3.50, 95% CI, 2.76–4.43) in the present study may support this finding. In the Kubo et al. study, patients with pulmonary MAC infection with no evidence of predisposing lung disease could lead to air trapping distal to the small airways [28] and bronchiectasis [10]. Meanwhile, Yamazaki et al. suggested that small airway dysfunction in MAC Infection without predisposing lung disease relating to the inflammation is probably related to the neutrophil [39]. These findings imply the NTM infection without comorbid disease is still critical to the development of RF [31].

The strength of this study is using a nationwide population data to perform the longitudinal assessment of RF risk in patients with NTM disease. These findings can be generalized to the general population. However, limitations must be considered when interpreting these findings. The insurance claims data provided no detailed information on smoking, body mass index, chest wall deformities, and environmental exposures. We were unable to assess how these factors are associated with the development of RF for patients with NTM in this study. However, we have performed an additional data analysis with a much smaller sample size for the non-NTM cohort (N = 3864), frequency matched by socio-demographic factors and comorbidities, including COPD as well (Table S3 in File S1). The results showed that the incidence of RF was 2.08-fold higher in the NTM cohort than in the non-NTM cohort with a HR of 2.27 (95% CI: 1.98–2.60) (Table S4 in File S1), indicating the impact of NTM remains strong. It is possible that disease diagnoses may be mistakenly coded in the claims data. To avoid misclassification, only patients with two diagnoses in 12 months were considered in this study. Meanwhile, the major types of infections caused by NTM included isolated pulmonary infection and pleurisy (59.5%) [40]. Study also found that few NTM patients with culture-positive specimens are clinical significant for RF in practice [41]. Patients with NTM infection without medical care could be excluded from the NTM cohort and under estimated the hazards in this study. These finding may need further intervention in future.

## Conclusion

In this nationwide study, we followed a large number of inpatients with nontuberculous mycobacterial infection with a mean follow-up of 4 years. These patients have a HR of 3.11 to develop respiratory failure compared with the general population. The risk is much greater in the first 6 months post diagnosis. The incidence of respiratory failure increases further for patients with other comorbidities particularly for those with chronic obstructive pulmonary disease and diabetes. Patients with mycobacterium avium complex are also at much higher risk of respiratory failure. These findings suggest that an early diagnosis of nontuberculous mycobacterial disease may assist in preventing respiratory failure.

## Supporting Information

File S1 **Appendix tables.** Table S1, Interaction between non-TB mycobacterial infection and comorbidity. Table S2, Incidence of respiratory failure events in study cohorts identified within 1 year and longer after diagnosis of non-TB mycobacterial infection and corresponding hazard ratio. Table S3, Patients of non-TB mycobacterial infection cohort and comparison subjects without non-TB mycobacterial infection frequency matched by sex, age, index year and comorbidities. Table S4, Incidence and adjusted hazard ratio of respiratory failure for patients with non-TB mycobacterial infection compared with subjects without non-TB mycobacterial infection.
(DOC)

## References

[1] Billinger ME, Olivier KN, Viboud C, de Oca RM, Steiner C, et al (2009) Nontuberculous mycobacteria-associated lung disease in hospitalized persons, United States, 1998–2005. Emerg Infect Dis 15: 1562–1569. 19861046 10.3201/eid1510.090196 PMC2866394

[2] Ringshausen FC, Apel RM, Bange FC, de Roux A, Pletz MW, et al (2013) Burden and trends of hospitalisations associated with pulmonary non-tuberculous mycobacterial infections in Germany, 2005–2011. BMC Infect Dis 13: 231. 23692867 10.1186/1471-2334-13-231 PMC3667050

[3] Huang WC, Chiou CS, Chen JH, Shen GH (2010) Molecular epidemiology of Mycobacterium abscessus infections in a subtropical chronic ventilatory setting. J Med Microbiol 59: 1203–1211. 20616186 10.1099/jmm.0.020586-0

[4] Shu CC, Lee CH, Wang JY, Jerng JS, Yu CJ, et al (2008) Nontuberculous mycobacteria pulmonary infection in medical intensive care unit: the incidence, patient characteristics, and clinical significance. Intensive Care Med 34: 2194–2201. 18648768 10.1007/s00134-008-1221-6

[5] Ringshausen FC, de Roux A, Pletz MW, Hamalainen N, Welte T, et al (2013) Bronchiectasis-associated hospitalizations in Germany, 2005–2011: a population-based study of disease burden and trends. PLoS ONE 8: e71109. 23936489 10.1371/journal.pone.0071109 PMC3731262

[6] Wallace JM, Hannah JB (1988) Pulmonary disease at autopsy in patients with the acquired immunodeficiency syndrome. West J Med 149: 167–171. 3266812 PMC1026365

[7] Huang JH, Kao PN, Adi V, Ruoss SJ (1999) Mycobacterium avium-intracellulare pulmonary infection in HIV-negative patients without preexisting lung disease: diagnostic and management limitations. Chest 115: 1033–1040. 10208205 10.1378/chest.115.4.1033

[8] Kim TS, Koh WJ, Han J, Chung MJ, Lee JH, et al (2005) Hypothesis on the evolution of cavitary lesions in nontuberculous mycobacterial pulmonary infection: thin-section CT and histopathologic correlation. AJR Am J Roentgenol 184: 1247–1252. 15788605 10.2214/ajr.184.4.01841247

[9] Jeong YJ, Lee KS, Koh WJ, Han J, Kim TS, et al (2004) Nontuberculous mycobacterial pulmonary infection in immunocompetent patients: comparison of thin-section CT and histopathologic findings. Radiology 231: 880–886. 15118112 10.1148/radiol.2313030833

[10] Moore EH (1993) Atypical mycobacterial infection in the lung: CT appearance. Radiology 187: 777–782. 8497629 10.1148/radiology.187.3.8497629

[11] Sue DYLD, editor (2008) Respiratory failure. 3rd ed. New York, NY: Lange Medical Books/McGraw Hill. 247–313 p.

[12] Kohno S, Seki M, Takehara K, Yamada Y, Kubo K, et al (2013) Prediction of requirement for mechanical ventilation in community-acquired pneumonia with acute respiratory failure: a multicenter prospective study. Respiration 85: 27–35. 22343936 10.1159/000335466

[13] Behrendt CE (2000) Acute respiratory failure in the United States: incidence and 31-day survival. Chest 118: 1100–1105. 11035684 10.1378/chest.118.4.1100

[14] Roussos C, Koutsoukou A (2003) Respiratory failure. Eur Respir J Suppl 47: 3s–14s. 10.1183/09031936.03.00038503 14621112

[15] Gong MN, Thompson BT, Williams P, Pothier L, Boyce PD, et al (2005) Clinical predictors of and mortality in acute respiratory distress syndrome: potential role of red cell transfusion. Crit Care Med 33: 1191–1198. 15942330 10.1097/01.ccm.0000165566.82925.14

[16] Lewandowski K (2003) Contributions to the epidemiology of acute respiratory failure. Crit Care 7: 288–290. 12930552 10.1186/cc2352 PMC270706

[17] Franca SA, Toufen C Jr, Hovnanian AL, Albuquerque AL, Borges ER, et al.. (2011) The epidemiology of acute respiratory failure in hospitalized patients: a Brazilian prospective cohort study. J Crit Care 26: 330 e331–338. 10.1016/j.jcrc.2010.10.010 21106336

[18] Orsini J, Ahmad N, Butala A, Flores R, Tran T, et al (2013) Etiology and Outcome of Patients with HIV Infection and Respiratory Failure Admitted to the Intensive Care Unit. Interdiscip Perspect Infect Dis 2013: 732421. 24065988 10.1155/2013/732421 PMC3771454

[19] Erasmus JJ, McAdams HP, Farrell MA, Patz EF Jr (1999) Pulmonary nontuberculous mycobacterial infection: radiologic manifestations. Radiographics 19: 1487–1505. 10555671 10.1148/radiographics.19.6.g99no101487

[20] Diagnosis and treatment of disease caused by nontuberculous mycobacteria. This official statement of the American Thoracic Society was approved by the Board of Directors, March 1997. Medical Section of the American Lung Association. Am J Respir Crit Care Med 156: S1–25. 9279284 10.1164/ajrccm.156.2.atsstatement

[21] Griffith DE, Aksamit T, Brown-Elliott BA, Catanzaro A, Daley C, et al (2007) An official ATS/IDSA statement: diagnosis, treatment, and prevention of nontuberculous mycobacterial diseases. Am J Respir Crit Care Med 175: 367–416. 17277290 10.1164/rccm.200604-571ST

[22] Andrejak C, Nielsen R, Thomsen VO, Duhaut P, Sorensen HT, et al (2013) Chronic respiratory disease, inhaled corticosteroids and risk of non-tuberculous mycobacteriosis. Thorax 68: 256–262. 22781123 10.1136/thoraxjnl-2012-201772

[23] Booth CM, Rapoport B (2011) Uptake of novel medical therapies in the general population. Curr Oncol 18: 105–108. 21655155 10.3747/co.v18i3.858 PMC3108862

[24] Booth CM, Mackillop WJ (2008) Translating new medical therapies into societal benefit: the role of population-based outcome studies. JAMA 300: 2177–2179. 19001630 10.1001/jama.300.18.2177

[25] Carol J, Buck BMSCPC, editor (2001) ICD-9-CM Volumes 1, 2, 3 and 2001 HCPCS. Philadelphia: W.B. Saunders: 298–299.

[26] Lee MR, Yang CY, Chang KP, Keng LT, Yen DH, et al (2013) Factors associated with lung function decline in patients with non-tuberculous mycobacterial pulmonary disease. PLoS ONE 8: e58214. 23483998 10.1371/journal.pone.0058214 PMC3590167

[27] Miravitlles M, Espinosa C, Fernandez-Laso E, Martos JA, Maldonado JA, et al (1999) Relationship between bacterial flora in sputum and functional impairment in patients with acute exacerbations of COPD. Study Group of Bacterial Infection in COPD. Chest 116: 40–46. 10424501 10.1378/chest.116.1.40

[28] Kubo K, Yamazaki Y, Masubuchi T, Takamizawa A, Yamamoto H, et al (1998) Pulmonary infection with Mycobacterium avium-intracellulare leads to air trapping distal to the small airways. Am J Respir Crit Care Med 158: 979–984. 9731034 10.1164/ajrccm.158.3.9802042

[29] Lee AR, Lee J, Choi SM, Seong MW, Kim SA, et al (2013) Phenotypic, immunologic, and clinical characteristics of patients with nontuberculous mycobacterial lung disease in Korea. BMC Infect Dis 13: 558. 24274658 10.1186/1471-2334-13-558 PMC4222821

[30] Koh WJ, Kwon OJ, Lee KS (2002) Nontuberculous mycobacterial pulmonary diseases in immunocompetent patients. Korean J Radiol 3: 145–157. 12271159 10.3348/kjr.2002.3.3.145 PMC2714412

[31] Prince DS, Peterson DD, Steiner RM, Gottlieb JE, Scott R, et al (1989) Infection with Mycobacterium avium complex in patients without predisposing conditions. N Engl J Med 321: 863–868. 2770822 10.1056/NEJM198909283211304

[32] Systrom DM, Wittram C (2005) Case records of the Massachusetts General Hospital. Case 9-2005. A 67-year-old man with acute respiratory failure. N Engl J Med 352: 1238–1246. 15788501 10.1056/NEJMcpc059003

[33] Shu CC, Lee CH, Hsu CL, Wang JT, Wang JY, et al (2011) Clinical characteristics and prognosis of nontuberculous mycobacterial lung disease with different radiographic patterns. Lung 189: 467–474. 21956280 10.1007/s00408-011-9321-4

[34] Yeh JJ, Wang YC, Sung FC, Chou CY, Kao CH. (2014) Nontuberculosis Mycobacterium Disease is a Risk Factor for Chronic Obstructive Pulmonary Disease: A Nationwide Cohort Study. Lung. [Epub ahead of print]. 10.1007/s00408-014-9574-9 24691889

[35] Shaaban H, Layne T, Sensakovic JW, Boghossian J (2013) Mycobacterium kansasii septicemia in an AIDS patient complicated by acute respiratory distress syndrome and acute liver failure. Int J STD AIDS. 10.1177/0956462413496080 23970643

[36] Babalik A, Kuyucu T, Ordu EN, Ernam D, Partal M, et al (2012) Non-tuberculous mycobacteria infection: 75 cases. Tuberk Toraks 60: 20–31. 22554363 10.5578/tt.2543

[37] Kazumi Y, Murase Y, Ishii K, Maeda S (2013) [The outbreak of nosocomial infection by Mycobacterium cheolnae chemovar niacinogenes and the cause of its spread]. Kansenshogaku Zasshi 87: 424–430. 23984591 10.11150/kansenshogakuzasshi.87.424

[38] Zheng C, Fanta CH (2013) Non-tuberculous mycobacterial pulmonary infection in the immunocompetent host. QJM 106: 307–315. 23365141 10.1093/qjmed/hct022

[39] Yamazaki Y, Kubo K, Fujimoto K, Matsuzawa Y, Sekiguchi M, et al (2000) Pulmonary function tests of Mycobacterium avium-intracellulare infection: correlation with bronchoalveolar lavage fluid findings. Respiration 67: 46–51. 10705262 10.1159/000029462

[40] Ding LW, Lai CC, Lee LN, Hsueh PR (2006) Disease caused by non-tuberculous mycobacteria in a university hospital in Taiwan, 1997–2003. Epidemiol Infect 134: 1060–1067. 16492317 10.1017/S0950268805005698 PMC2870472

[41] Simons S, van Ingen J, Hsueh PR, Van Hung N, Dekhuijzen PN, et al (2011) Nontuberculous mycobacteria in respiratory tract infections, eastern Asia. Emerg Infect Dis 17: 343–349. 21392422 10.3201/eid1703100604 PMC3165997

