# Supplementary material for: Nontuberculous Mycobacterial Infection Is Associated with Increased Respiratory Failure: A Nationwide Cohort Study
Source: PLoS One. 2014 Jun 11;9(6):e99260. doi: 10.1371/journal.pone.0099260 (PMC4053398; doi:10.1371/journal.pone.0099260)
Supplement: File S1 — Appendix tables. Table S1, Interaction between non-TB mycobacterial infection and comorbidity. Table S2, Incidence of respiratory failure events in study cohorts identified within 1 year and longer after diagnosis of non-TB mycobacterial infection and corresponding hazard ratio. Table S3, Patients of non-TB mycobacterial infection cohort and comparison subjects without non-TB mycobacterial infection frequency matched by sex, age, index year and comorbidities. Table S4, Incidence and adjusted hazard ratio of respiratory failure for patients with non-TB mycobacterial infection compared with subjects without non-TB mycobacterial infection. (DOC) [file pone.0099260.s001.doc]

| Appendix table 1. Interaction between non-TB mycobacterial infection and comorbidity. | | | |
| --- | --- | --- | --- |
| Variables | Comorbidity | *p value*^#^ |  |
| Non-TB mycobacterial infection | Tuberculosis | 0.41 |  |
| Non-TB mycobacterial infection | Bronchopneumonia | 0.32 |  |
| Non-TB mycobacterial infection | Pneumonia | 0.07 |  |
| Non-TB mycobacterial infection | COPD | 0.008 |  |
| Non-TB mycobacterial infection | Pneumoconiosis | 0.87 |  |
| Non-TB mycobacterial infection | Diabetes | 0.0007 |  |
| Non-TB mycobacterial infection | Liver cirrhosis | 0.22 |  |
| Non-TB mycobacterial infection | Cancer | 0.61 |  |
| Non-TB mycobacterial infection | ESRD | 0.49 |  |
| Non-TB mycobacterial infection | Malnutrition | 0.29 |  |
| Non-TB mycobacterial infection | HIV | 0.96 |  |
| Model adjusted for age, sex. ; ^#^*p*-value for interaction. | | | |

| Appendix table 2. Incidence of respiratory failure events in study cohorts identified within 1 year and longer after diagnosis of non-TB mycobacterial infection and corresponding hazard ratio | | | | | | | | | |  |  | |  |
| --- | --- | --- | --- | --- | --- | --- | --- | --- | --- | --- | --- | --- | --- |
|  | Without non-TB mycobacterial infection | | | With non-TB mycobacterial infection | | |  |  |  | | |  | |
| Follow time | Event | PY | Rate^#^ | Event | PY | Rate^#^ | IRR^＊^(95% CI) | Adjusted HR^†^ | Log-Rank test | | |  | |
|  |  |  |  |  |  |  |  | (95% CI) | *p value* | | |  | |
| <1years | 162 | 15205 | 10.65 | 284 | 3420 | 83.0 | 7.79(7.15-8.50) | 5.31(4.26-6.63) | <.0001 | | |  | |
| ≥1 years | 513 | 50957 | 10.07 | 303 | 9916 | 30.6 | 3.04(2.79-3.30) | 2.33(1.97-2.75) | <.0001 | | |  | |
| PY, person-years; Rate^#^, incidence rate, per 1,000 person-years; IRR^＊^, incidence rate ratio; CI, confidence interval; Adjusted HR^†^ : multivariable analysis included age, sex, income, occupation, and comorbidities. | | | | | | | | | | | | | |

| Appendix table 3. Patients of non-TB mycobacterial infection cohort and comparison subjects without non-TB mycobacterial infection frequency matched by sex, age, index year and comorbidities. | | | | | |
| --- | --- | --- | --- | --- | --- |
|  | Non-TB mycobacterial infection | | | |  |
|  | No(N= 3822) | | Yes(N= 3864) | |  |
| Variables | n | % | n | % | *p*-value |
| Sex^†^ |  |  |  |  | 0.95 |
| Women | 1439 | 37.7 | 1454 | 37.6 |  |
| Men | 2383 | 62.4 | 2410 | 62.4 |  |
| Age, year^†^ |  |  |  |  | 0.90 |
| <65 | 2410 | 63.1 | 2347 | 60.7 |  |
| ≥65 | 1504 | 39.3 | 1517 | 39.3 |  |
| Mean (SD) ^#^ | 55.6(21.2) | | 55.7(21.1) | | 0.86 |
| Income, NTD^†^ |  |  |  |  | 0.18 |
| < 15000 | 1933 | 50.6 | 2017 | 52.2 |  |
| 15000-25000 | 1259 | 32.9 | 1264 | 32.7 |  |
| ≥ 25000 | 630 | 16.5 | 583 | 15.1 |  |
| Occupation^†^ |  |  |  |  | 0.61 |
| White collar | 1769 | 46.3 | 1801 | 46.6 |  |
| Blue collar | 1546 | 40.5 | 1523 | 39.4 |  |
| Others | 491 | 12.8 | 527 | 13.6 |  |
| Comorbidity^†^ |  |  |  |  |  |
| Tuberculosis | 872 | 22.8 | 900 | 23.3 | 0.62 |
| Bronchopneumonia | 140 | 3.66 | 154 | 3.99 | 0.46 |
| Pneumonia | 767 | 20.1 | 795 | 20.6 | 0.58 |
| COPD | 752 | 19.7 | 766 | 19.8 | 0.87 |
| Pneumoconiosis | 87 | 2.28 | 101 | 2.61 | 0.34 |
| Diabetes | 733 | 19.2 | 748 | 19.4 | 0.84 |
| Liver cirrhosis | 302 | 7.90 | 316 | 8.18 | 0.66 |
| Cancer | 346 | 9.05 | 363 | 9.39 | 0.60 |
| ESRD | 72 | 1.88 | 76 | 1.97 | 0.79 |
| Malnutrition | 47 | 1.23 | 59 | 1.53 | 0.26 |
| HIV | 173 | 4.53 | 186 | 4.81 | 0.55 |
| ^†^Chi-square test ; ^#^ Student’s t-test; In occupation variables missing 29 numbers (control:16; case:13). | | | | | |

| Appendix table 4. Incidence and adjusted hazard ratio of respiratory failure for patients with non-TB mycobacterial infection compared with subjects without non-TB mycobacterial infection. | | | | | | | | |
| --- | --- | --- | --- | --- | --- | --- | --- | --- |
| Non-TB mycobacterial infection | | | | | | Compared to comparisons | |  |
|  | No |  |  | Yes |  |  |
| Case | PY | Rate^#^ | Case | PY | Rate^#^ | IRR^＊^  (95% CI) | Adjusted HR^†^  (95% CI) |  |
| 325 | 15358 | 21.2 | 587 | 13337 | 44.0 | 2.08(1.85-2.34) | 2.27(1.98-2.60) |  |
| PY, person-years; Rate^#^, incidence rate, per 1,000 person-years; IRR^＊^, incidence rate ratio; CI, confidence interval; HR^†^: hazard ratio, p<0.001 | | | | | | | | |
